# Molecular cloning, expression analysis, and functional characterization of an interleukin-15 like gene in common carp (*Cyprinus carpio* L.)

**DOI:** 10.3389/fimmu.2024.1502847

**Published:** 2024-11-19

**Authors:** Xinyu Jiang, Xiaoyu Wang, Mengjie Gao, Xudong Li, Yi Ding, Yunjie Song, Hehe Xiao, Xianghui Kong

**Affiliations:** ^1^ Engineering Lab of Henan Province for Aquatic Animal Disease Control, College of Fisheries, Henan Normal University, Xinxiang, Henan, China; ^2^ Hangzhou Xiaoshan Donghai Aquaculture Co., Ltd, Hangzhou, Zhejiang, China; ^3^ College of Life Sciences, Henan Normal University, Xinxiang, Henan, China; ^4^ Fishery Technology Extension Station of Henan Province, Zhengzhou, Henan, China

**Keywords:** interleukin-15L, immune response, bioactivity analysis, *Cyprinus carpio*, phagocytosis, chemotaxis, leukocytes

## Abstract

Interleukin-15 (IL-15) is a crucial cytokine involved in immune system regulation, which is produced by various cell types, including dendritic cells, monocytes, and macrophages. IL-15 plays a key role in the proliferation and activation of natural killer (NK) cells, CD8^+^ T cells, and memory CD8^+^ T cells, supporting their survival and enhancing their effector functions. Although IL-15 homologues in fish have been identified, their functions remain poorly understood. In this study, we cloned and investigated the bioactivities of an IL-15 homologue, referred to as IL-15 like (*Cc*IL-15L), in common carp (*Cyprinus carpio* L.). An expression pattern analysis revealed that *CcIL-15L* was constitutively expressed in all examined tissues of healthy common carp, with the highest expression level observed in the intestine. Additionally, *CcIL-15L* expression was significantly up-regulated in the head kidney, spleen, gills, and intestine following *Aeromonas hydrophila* infection. *In vitro*, the recombinant protein *Cc*IL-15L can significantly up-regulated the gene expression levels of pro-inflammatory cytokines (*IL-1β*, *IL-6*, *IFN-γ*, and *TNF-α*) and NK cell activation (*perforin* and *Eomesa*). We constructed a 3×FLAG eukaryotic expression vector and successfully expressed it in common carp by intramuscular injection. Additionally, the heterologous *Cc*IL-15L protein was successfully overexpressed *in vivo*, and immune-related genes including *CD4-1*, *CD8β2*, *TNF-α*, and *IgM* showed significant induction in the head kidney and spleen. Furthermore, *Cc*IL-15L overexpression reduced the bacterial loads after 24 h post-*A. hydrophila* infection in the liver, spleen, and kidney. Phagocytic and chemotaxis assays showed that r*Cc*IL-15L could promoted the phagocytosis and chemotactic abilities of common carp HKLs. Our study provides a new perspective on the role for *Cc*IL-15L in immunological functions in common carp.

## 1 Introduction

Interleukin-15 (IL-15) is a pleiotropic cytokine influencing both innate and adaptive immunity. It is a member of the IL-2 cytokine family, which includes IL-2, IL-4, IL-7, IL-9, and IL-21 (1). These cytokines are collectively termed the γc family due to their shared γc receptor subunit (2). IL-15 is essential for the development, survival, and activation of memory CD8+ T cells, natural killer (NK) cells, NKT cells, and γδ T cells (3–6). Though structurally similar to IL-2, IL-15 has minimal sequence overlap with IL-2 (7) and is primarily secreted by T cells, monocytes/macrophages, dendritic cells, NK cells, keratinocytes, fibroblasts, epithelial cells, and neurons (8). IL-15 expression can be upregulated in response to inflammatory stimuli and infections (9, 10).

In mammals, IL-15 signaling involves a trimeric receptor complex, composed of the IL-15R-specific subunit, the IL-2/IL-15Rβ subunit, and the γc receptor. The IL-2/IL-15Rβ and γc subunits recruit Janus kinases (JAK)-1 and JAK3, respectively, activating the JAK/STAT pathway, which leads to phosphorylation of STAT-3 and STAT-5 and subsequent nuclear translocation.

In teleosts, research on IL-15 homologs has predominantly centered on IL-15 itself, with limited investigation into IL-15-like (IL-15L) genes (8, 11–16). IL-15L was first identified in teleost fish (11, 17, 18) and later discovered in cartilaginous fish (19). To date, IL-15L genes have been identified in multiple fish species, including zebrafish (*Danio rerio*), fugu (*Takifugu rubripes*), rainbow trout (*Oncorhynchus mykiss*), and Atlantic salmon (*Salmo salar*) (11, 18, 20). Fish IL-15L is constitutively expressed across various tissues in healthy fish. Predicted signal peptides are found only in rohu IL-15, suggesting similar secretory pathways with human IL-15. Nonetheless, the biological role of IL-15L in fish remains understudied, with evidence of its immune functions limited to rainbow trout, where recombinant IL-15L protein induces type II immune responses, as shown by IL-4/13A, IL-4/13B1, and IL-4/13B2 upregulation (20).

The common carp (*Cyprinus carpio*) is a widely cultivated freshwater species with high ecological, cultural, and economic value. In this study, we identified an IL-15L gene in common carp and examined *CcIL-15L* expression under normal and *Aeromonas hydrophila* challenge conditions. *In vitro*, the effects of recombinant *Cc*IL-15L on pro-inflammatory cytokines (IL-1β, IL-6, IFN-γ, and TNF-α) and NK cell activation markers (perforin and Eomes) were investigated. *In vivo*, we assessed *Cc*IL-15L’s role in immune regulation and defense. This research clarifies *Cc*IL-15L gene characteristics and its function in fish immune response.

## 2 Materials and methods

### 2.1 Experimental fish

Common carp (an average weight of 50 ± 5 g) were purchased from a commercial fish farm in Xinxiang (Henan province, China). Before the experiment, the fish were kept in freshwater tanks at 25 ± 2 °C for 2 weeks, and fed with commercial pellets twice a day.

All animal experiments in this study were performed following the protocols of the “Guidelines for Experimental Animals” of the Ministry of Science and Technology (Beijing, China), and all experiments involving animals were approved by the Animal Care and Use Ethics Committee of the Henan Normal University.

### 2.2 Gene cloning of *Cc*IL-15L

The predicted IL-15L sequence of common carp was obtained based on the transcriptional database of common carp and the sequence of zebrafish IL-15 (NM_001039565.1). Subsequently, specific primers were designed based on the predicted gene sequence of IL-15L (**Table 1**). Total RNA of the liver from healthy common carp was extracted using TRIzol (TaKaRa, Japan), and then the first strand of cDNA was synthesized. The cDNA sequence of *Cc*IL-15L was amplified by polymerase chain reaction. The PCR products were then examined with 1.5% agarose gel, and purified with an agarose gel DNA purification kit (OMEGA, Bio-Tek). The target sequences were ligated into the pMD19-T vector and transformed into DH-5α for sequencing.

**Table 1:** Sequence of primer used in this study.

Primers	Sequence (5’→3’)	Application
IL-15L-F	ATGACAGATGTGTTTATGAGGGAGA	Verify the full length
IL-15L-R	TCAGATCATGTTTTTAATGTGCGTT	Verify the full length
rIL-15L-F	CCGGAATCCATGACAGATGTGTTTATGAGGGAGA (Ecor I)	Plasmid construction
rIL-15L-R	CCCAAGCTTGGGATCATGTTTTTAATGTGCGTT (Hind III)	Plasmid construction
pcIL-15L-F	CCGGAATTCCACCATGACAGATGTGTTTATGA (Ecor I)	Plasmid construction
pcIL-15L-R	CCCAAGCTTGGGATCATGTTTTTAATGTGCGTT (Hind III)	Plasmid construction
IL-15L-DistF	AGAGTGCGGCTAAATCTCAG	Real-time PCR
IL-15L-DistR	TTTCAGCATTCATCCGTTGT	Real-time PCR
TNF-α-DistF	GAGGATTGCTGCCCTTACCG	Real-time PCR
TNF-α-DistR	AAATGGATGGCTGCCTTGGA	Real-time PCR
IL-1β-DistF	AAGGAGGCCAGTGGCTCTGT	Real-time PCR
IL-1β-DistR	CCTGAAGAAGAGGAGGCTGTCA	Real-time PCR
Perforin-DistF	CGGCTGACTGGAAAGTTGGT	Real-time PCR
Perforin-DistR	GTTGCGGTAGGCGTCTGGAT	Real-time PCR
IL-6-DistF	GATTGGTACAACGAAGAAGA	Real-time PCR
IL-6-DistR	GCATGACCCATATATGACCCA	Real-time PCR
Eomesa-DistF	AGGCGGATGTTCCCATTTCT	Real-time PCR
Eomesa-DisR	TTGCATGTTATTGTCGGCTTT	Real-time PCR
IFNγ-DistF	TATGGGCGATCAAGGAAGAT	Real-time PCR
IFNγ-DistR	TTGTGATTCTGGCTTGTCGT	Real-time PCR
CD4-1-DistF	AACACCAAGAAACTTAGCAGGAA	Real-time PCR
CD4-1-DistR	AGGGAAGATGGATGAGGAGG	Real-time PCR
CD8β2-DistF	AAATCAACGGCTCGGAAACT	Real-time PCR
CD8β2-DistR	CAGGGTGTAGACCATCCTCTGT	Real-time PCR
IgM-DistF	CACAAGGCGGGAAATGAAGA	Real-time PCR
IgM-DistR	CTGATAAAGCTTTGCACTTCAGCA	Real-time PCR
CSF1RF	CTCGGGCAGCACAAGAACAT	Real-time PCR
CSF1RR	CCATCAGCCTCGCTATCCAA	Real-time PCR
Ah-F	GAAAGGTTGATGCCTAATACGTA	Verify the bacteria
Ah-R	CGTGCTGGCAAC AAA GGACAG	Verify the bacteria
EF-1αF	CAGCACAAACATGGGCTGGTTC	Real-time PCR
EF-1αR	ACGGGTACAGTTCCAATACCTCCA	Real-time PCR

Underlined nucleotides are restriction sites of the enzymes indicated in the brackets. Kozak sequences are represented in rectangle.

### 2.3 Analyses of sequence characteristics

*Cc*IL-15L open reading frames (ORFs) were identified using NCBI ORF finder (https://www.ncbi.nlm.nih.gov/orffinder/) and the deduced amino acid sequence was predicted with translate tool (https://web.expasy.org/translate/). SignalP (http://www.cbs.dtu.dk/services/SignalP) was used to predict signal peptides, while molecular weight and isoelectric point were calculated using online software (https://web.expasy.org/compute_pi/). ClustalW X2 was utilized for multiple sequence comparisons, and SMART (SMART, http://smart.emblheidelberg.de/) was employed to predict protein motif features. Phylogenetic analysis was performed using MEGA7.0 software with the neighbor-joining method and 10000 replicates were used to test branch reliability.

### 2.4 Tissues expression analysis of *Cc*IL-15L by real-time PCR

To investigate the expression pattern of *Cc*IL-15L in different tissues (liver, head kidney, spleen, muscle, intestine, brain, gills, and skin), five healthy common carp were immobilized by MS-222 anesthesia and sacrificed. Then the tissues were sampled and placed in enzyme-free EP tubes, respectively. Then, TRizol reagent (TaKaRa, Japan) was used to extract total RNA according to the instructions of the manufacturer. The quality of extracted RNA was detected by 1% agarose gel electrophoresis and the purity was determined by the NanoDrop 2000 spectrophotometer (Thermo Scientific, USA). Subsequently, the first strand of cDNA was synthesized according to Hifair^®^ II 1st Strand cDNA Synthesis SuperMix (Yeason, Shanghai, China) instructions. The primers are listed in **Table 1**. The RT-qPCR reaction system is 10 μL, including 0.2 μL of each primer, 2.6 μL of sterile ultrapure water, 2 μL of diluted cDNA template, and 5 μL of Hieff UNICON^®^ Universal Blue qPCR SYBR Green Master Mix (Yeason, Shanghai, China). The reaction system was carried out according to the following steps: 95°C for 2 min, followed by 40 cycles of 95°C for 10 s, 60°C for 30 s, and 72°C for 20 s. *EF-1α* was used as the internal reference gene. The relative mRNA expression levels of *Cc*IL-15L were calculated using the 2^-ΔΔCt^ method (21).

### 2.5 Bacterial challenge

The pathogen *A. hydrophila* was isolated from common carp and preserved in our laboratory, and they were prepared according to the previous studies (22). In brief, the bacteria were cultured in LB medium at 28°C for 12 h with constant shaking (200 rpm), and then they were centrifuged at 6000 rpm for 10 min and washed twice with PBS. The fish were randomly selected for the challenge experiment. Each common carp was intraperitoneal injected (i.p.) with 100 μL inoculum (approximately 1.5×10^6^ CFU/ml). Three common carp were randomly collected each time at 0, 6, 12, 24, and 48 h post-injection (hpi). After that, the gills, intestine, spleen, and head kidney of common carp were sampled. The RT-qPCR assay was then performed as described above. The experiments were carried out in triplicate.

### 2.6 Production and purification of recombinant proteins

To construct a prokaryotic expression vector, the *Cc*IL-15L was ligated into pET-32a (+) plasmids. The successfully constructed pET-32a-IL-15L was transferred into component cells of *Escherichia coli* BL21 (Biomed, China). The cells were cultured at 37°C until OD_600_ reached 0.6, and then the recombinant proteins were induced with 1.0 mM IPTG at 37°C overnight and detected with 12.5% SDS-PAGE (Polyacrylamide gel electrophoresis). After that, the rIL-15L was lysed using 6 M urea and purified by NI-NTA nitrilotriacetic acid (NI-NTA). The dialysis tubing was first boiled in water for 15 min and then rinsed with sterile water. One end of the tubing was sealed with a clip, and the protein solution was added from the other end. The tubing was then placed in a dialysis solution at 4°C. The dialysis solution was changed every 6 h for a total of five times, with urea concentrations of 6 M, 4 M, 2 M, 1 M, and PBS, respectively. After dialysis, the protein concentration and purity were assessed. Purity was evaluated by SDS-PAGE, and protein concentration was determined using the BCA method. The protein was concentrated to 1 mg/mL using an ultrafiltration tube and stored at -80°C for future use. The thioredoxin was used as the a control protein apart from PBS. The proteins were then analyzed by 12.5% SDS-PAGE and the protein concentration was determined by the BCA method.

### 2.7 Detection of mRNA expression levels of the immune-related genes in head kidney leukocytes treated by r*Cc*IL-15L

According to a previous study (22), primary head kidney leukocytes (HKLs) were separated from the head kidney of healthy common carp. In brief, head kidneys were collected from the fish, and they were placed on a 70 μm nylon mesh, and gently pushed through the mesh with constant dripping of cold L15 medium containing 0.1% FBS, 0.1% heparin, 100 U/mL penicillin, and 100 U/ml streptomycins. The cell suspension was centrifuged at 400 × *g* for 30 min on a 45% percoll gradient. The cells on the medium-percoll interface were collected by centrifugation at 400 × g at 4°C for 10 min and washed twice with the medium. The cells were seeded into 6-well cell culture plates at 1.5 × 10^6^ cells/well, and cultured at 28°C in a 5% CO_2_ incubator for 2 h (23). After 2 h of cultivation, different concentrations of r*Cc*IL-15L (2, 20, and 200 ng/mL) were added to the wells and incubated for 12 h, and the wells were added with PBS were set as the control group. The mRNA expression levels of IL-1β, TNF-α, IFN-γ, IL-6, perforin, and Eomesa were then examined by RT-qPCR. The primers were listed in **Table 1**.

### 2.8 *In vivo* overexpression of *Cc*IL-15L and its effect on inflammatory cytokine expression

The specific primers of eukaryotic expression plasmids (**Table 1**) were designed based on genes of *Cc*IL-15L, and added Kozak sequences. The purified PCR products of *Cc*IL-15L and expression vector pcDNA™3.1/3×FLAG were digested with the restriction enzymes *EcoR* I and *Hind* III and ligated with the T4 DNA Ligase (TaKaRa, Japan). Then, the linked products were transformed into *E. coli* DH-5α, which were then plated on LB-ampicillin media and were grown overnight at 37°C. The transformants were verified by sequencing and double enzymatic digestion. The successfully constructed plasmid is named pcIL-15L. Plasmid DNA was extracted using Endo-Free plasmid Kit (Tiangen, China), and removed the endotoxin.

Common carp were assigned randomly into three groups and subjected to intramuscular injection of 100 μl of pcIL-15L (200 μg/ml), 100 μl of the empty pcDNA 3.1 vector (pcN3) (200 μg/ml), or 100 μl of PBS. On fifth day after plasmid administration, the head kidney and spleen samples were collected from three fish in each group. The expression levels of immune-related genes (*CD4-1*, *CD8β2*, *TNF-α*, and *IgM*) were assessed using RT-qPCR as described above. The primer sequences used in this study are provided in **Table 1**. The plasmid was introduced into common carp via intramuscular injection, and the overexpression of *Cc*IL-15L was confirmed through RT-qPCR, western blotting, and immunofluorescence assay (IIFA) at 5 d post plasmid administration.

### 2.9 The effect of *in vivo* overexpression of *Cc*IL-15L on resistance to *A. hydrophila* infection

Five days after introducing the recombinant plasmid into the fish using the aforementioned method, each fish was intraperitoneally injected with 0.2 mL of *A. hydrophila* at a concentration of 5×10^6^ CFU/mL. After 12 and 24 h, the liver, spleen, and kidney were aseptically collected. The tissues were homogenized and diluted with sterile PBS, then spread in triplicate on LB agar plates. The plates were incubated at 28°C for 24 h, and the number of colonies was counted. Each colony was verified using PCR with *A. hydrophila*-specific primers (**Table 1**).

### 2.10 Phagocytic and chemotaxis assays

1× 10^6^ HKLs in RPMI 1640 medium supplemented with 10% FBS were seeded into 24-well plates and incubated overnight at 28°C with 5% CO_2_. The r*Cc*IL-15L was added to the cultures with the final dose 200 ng/mL for 24 h. Fluorescent latex beads were then introduced at a 1:25 cell-to-bead ratio, as previously described (24). After removing non-ingested beads using BSA and D-glucose buffer, phagocytic activity was analyzed via flow cytometry (24).

The chemotaxis assays were conducted using transwell chambers (Corning) with 13 mm filters and a pore size of 8 µm. The lower chambers were filled with r*Cc*IL-15L (500 ng/mL) or supernatants from HKLs treated with r*Cc*IL-15L for 24 h. Common carp HKLs (1 × 10^6^ cells/well) were added to the upper chambers. After a 4-hours incubation, migrated HKLs in the bottom wells were quantified under an Axio Observer Z1 microscope (Zeiss, Germany) in 2 min. Chemotactic activity was assessed by calculating the chemotactic index, defined as the ratio of cells migrating in response to r*Cc*IL-15L or supernatants from HKLs treated with r*Cc*IL-15L compared to the number of cells that migrated by PBS, which served as the negative control. Total RNA of the migrated cells were extracted as described above, and the expression levels of different cell type marker genes were detected using RT-qPCR. The marker gene primers were listed in **Table 1**.

### 2.11 Histopathological examination

Common carp were administered with pcIL-15L, pcN3, or PBS as above. After 5 d post-plasmid administration, common carp were injected intramuscularly with 100 μl *A. hydrophila* (approximately 1.5×10^6^ CFU/ml). At 24 hpi, the head kidney and spleen were removed and placed with 1 ml of 4% paraformaldehyde, and then submerged in 4% paraformaldehyde overnight. The tissues were embedded in paraffin, sectioned, and stained with HE and the images were captured and processed with ZEN/ZEN lite imaging software from Zeiss.

### 2.12 Statistical analysis

Data was shown as mean + standard error of the mean (SEM). Statistical significance was analyzed with one-way analysis of variance (ANOVA), implemented in software GraphPad Prism 8. Statistical significance was set as *p* < 0.05 (*) and *p* < 0.01 (**).

## 3 Results

### 3.1 Sequence analysis of *Cc*IL-15L

**Figure 1:**
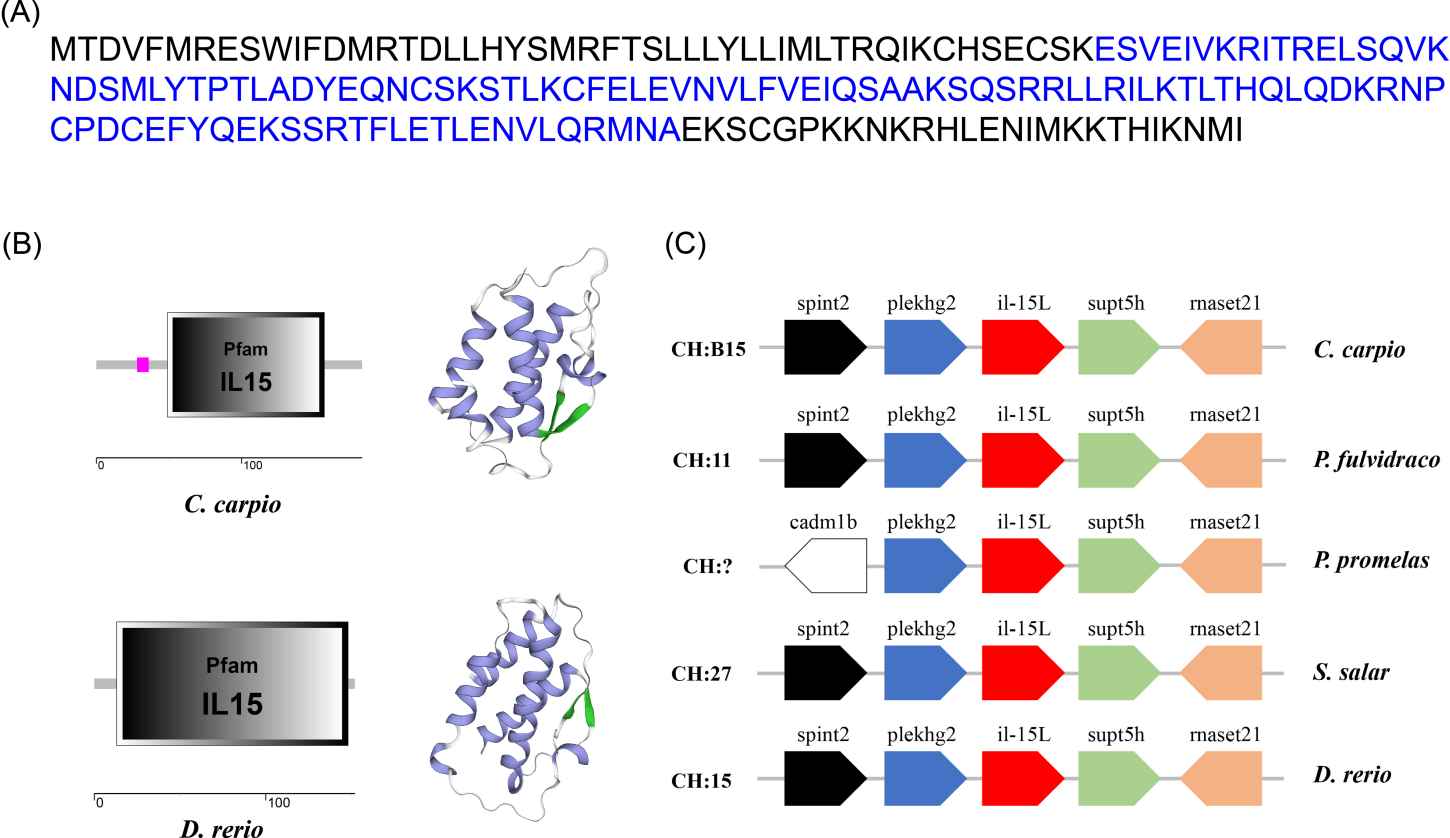
**(A)** The protein sequence **(A)**, domain organization **(B)**, 3D structural **(B)**, and gene synteny **(C)** of *Cc*IL-15L. **(A)** The protein sequence of *Cc*IL-15L. The IL-15 domain is shown in blue font. **(B)** The zebrafish and common carp IL-15L domain organization were predicted by SMART. 3D structural model of IL-15L was predicted in zebrafish and common carp using SWISS-MODEL server (α-helix in purple, β-sheet in green, and white in random curls). **(C)** The synteny information of the IL-15L genes from common carp, yellow catfish, fathead minnow, rainbow trout, and zebrafish. Arrows indicate gene transcription orientation.

**Figure 2:**
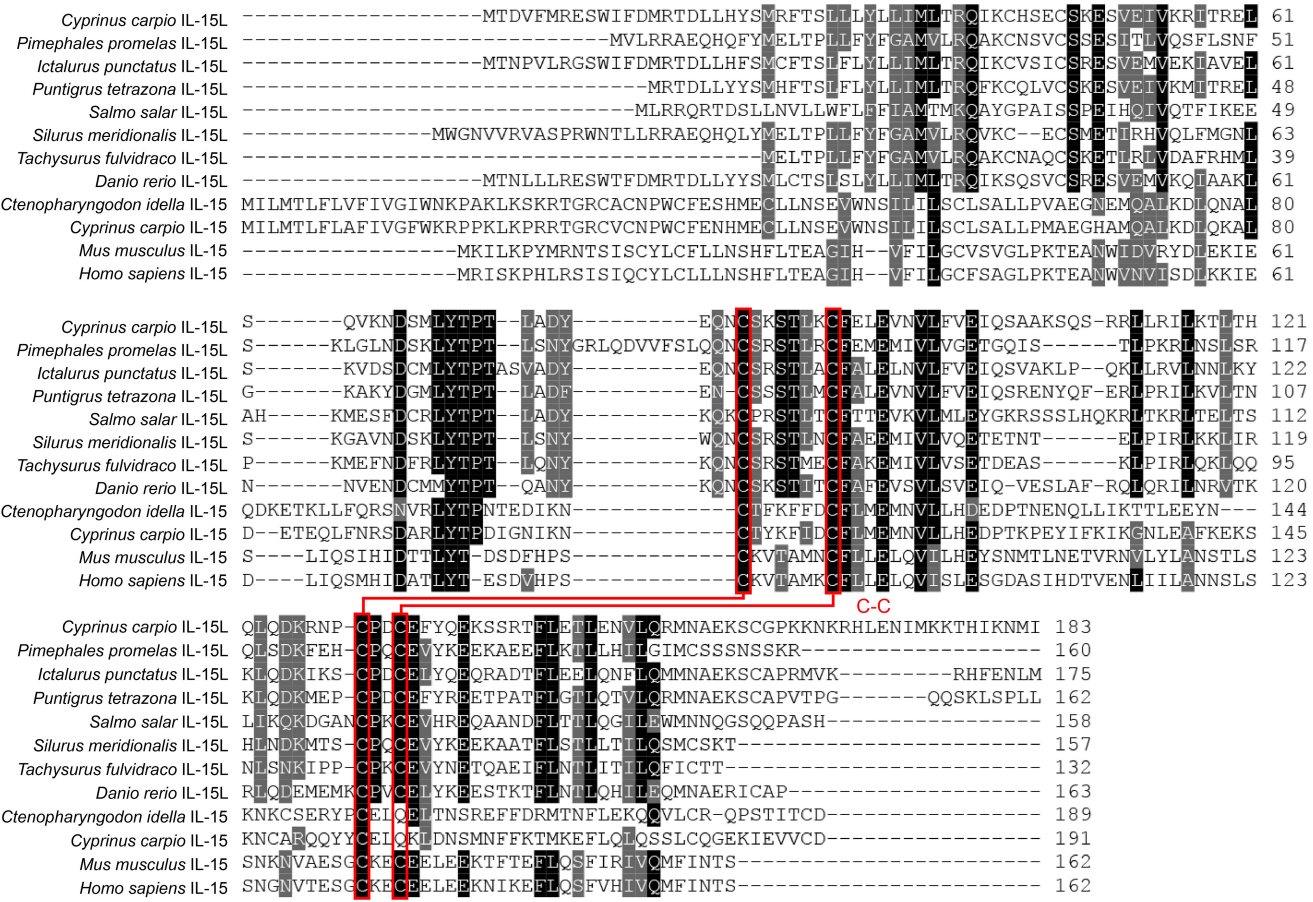
Multiple alignment of amino acid sequences of IL-15 and IL-15L. Four conserved cysteine residues are indicated in red font, which can form two potential intrachain disulfide bridges and are paired with lines (5).

**Figure 3:**
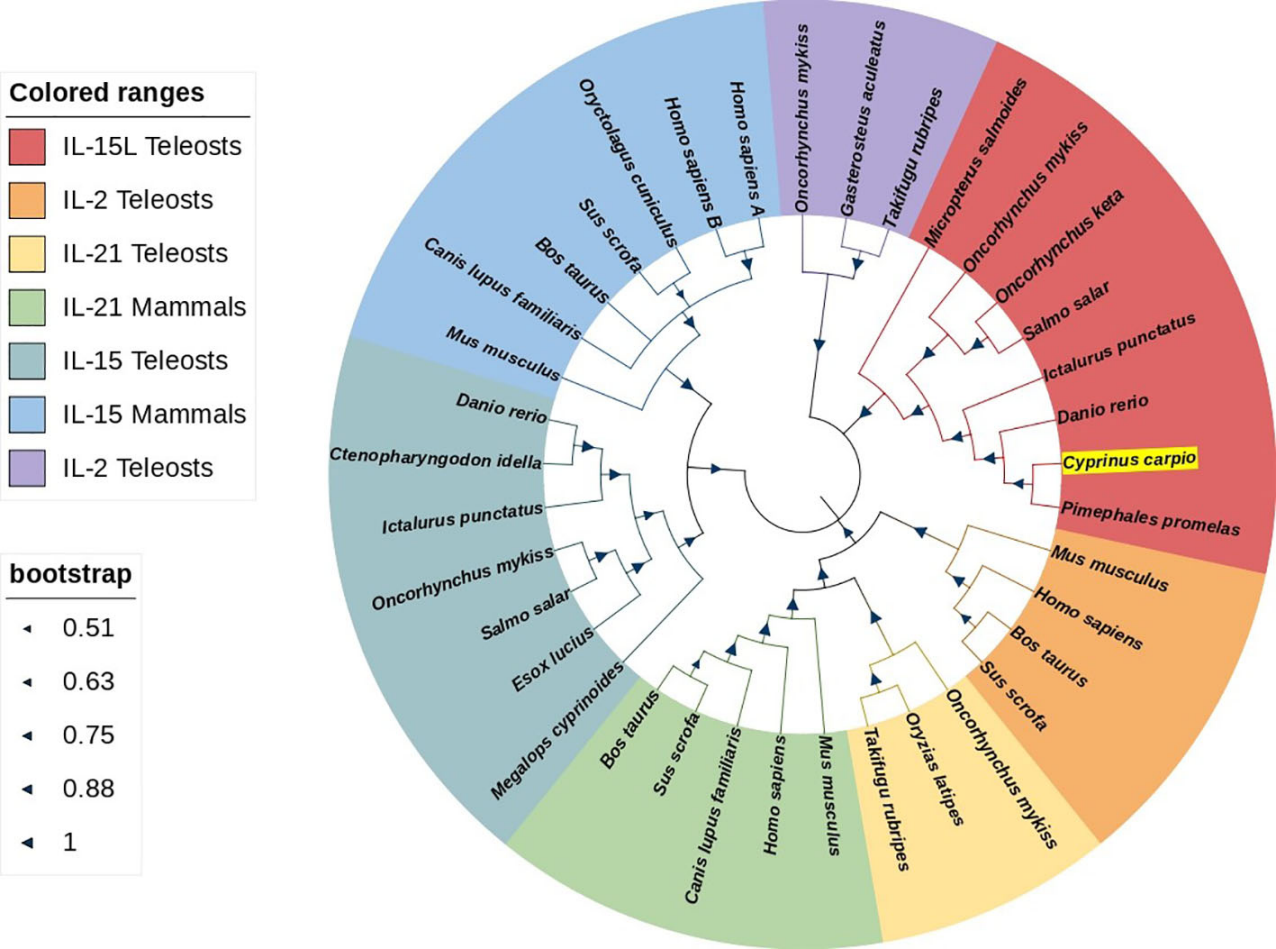
Phylogenetic tree analysis of IL-15 homologues, IL-2, and IL21. *Cc*IL-15L is shown in highlight by yellow. The tree was constructed using Neighbor-Joining method within the MEGA-7.0 program. The percentage of bootstrap values represent the confidence of bootstrap test with 10000 replicates. The GenBank accession numbers of amino acid sequences used here are as follows: for IL-15L: *Micropterus salmoides*, XP_038549918.1; *Oncorhynchus mykiss*, CDM74103.1; *Oncorhynchus keta*, XP_035627921.1; *Salmo salar*, XP_045580359.1; *Ictalurus punctatus*, XP_017345806.1; *Danio rerio*, XP_005157574.1; *Pimephales promelas*, XP_039527011.1; for IL-2: *Mus musculus*, NP_032392.1; *Homo sapiens*, NP_000577.2; *Bos taurus*, NP_851340.2; Sus scrofa, NP_999026.1; *Oncorhynchus mykiss*, NP_001157537.1; *Gasterosteus aculeatus*, NP_001254611.1; *Takifugu rubripes*, NP_001033083.1; for IL-21: *Oncorhynchus mykiss*, NP_001233260.1; *Oryzias latipes*, NP_001121987.1; *Takifugu rubripes*, NP_001033082.1; *Mus musculus*, NP_001277970.1; *Homo sapiens*, NP_001193935.1; *Canis lupus*, BAD22569.1; *Sus scrofa*, NP_999580.1; *Bos taurus*, BAC87747.1; for IL-15: *Megalops cyprinoides*, XP_036407763.1; *Esox Lucius*, XP_010886628.1; *Salmo salar*, NP_001265994.1; *Oncorhynchus mykiss*, XP_021430745.2; *Ictalurus punctatus*, XP_017319088.1; *Ctenopharyngodon Idella*, QCE20959.1; *Danio rerio*, NP_001034654.2; *Mus musculus*, AAH23698.1; *Canis lupus*, NP_001184117.1; *Bos taurus*, AAA85130.1; Sus scrofa, AAB72031.1; *Oryctolagus cuniculus*, AAZ82803.1; *Homo sapiens* IL-15A, NP_000576.1; *Homo sapiens* IL-15B, NP_751915.1.

The ORF of *Cc*IL-15L was 552 nucleotides (NCBI accession number: OQ981449), encoding a protein 183 amino acids (aa) without a predicted signal peptide (**Figure 1A**) and with a theoretical molecular weight (MW) of 21.7 kDa and a theoretical PI of 9.17. The deduced amino acid sequence indicated an IL-15 domain at residue 49–157, which was similar to zebrafish (**Figure 1B**). In addition, the IL-15L protein tertiary structures of common carp and zebrafish were predicted by SWISS-Model, and the results showed that *Cc*IL-15L was highly similar to the tertiary structures of IL-15L from zebrafish (**Figure 1B**). Syntenic analysis showed that common carp and other teleost IL-15L are located in the same locus adjacent *plekhg2* and *supt5h* (**Figure 1C**). Homology comparison showed that the *Cc*IL-15L shared sequence identity with those of other fish species (**Table 2**). *Cc*IL-15L indicated the highest sequence identity with Fathead minnow (*Pimephales promelas*) IL-15L (64.3%) and Tiger barb (*Puntigrus tetrazona*) IL-15L (60.1%), and the lowest sequence identity with grass carp (*Ctenopharyngodon idellus*) IL-15 (13.9%). Multiple alignment of IL-15L and IL-15 aa sequences showed that four cysteine residues were conserved between fish and mammals (**Figure 2**). Four cysteine (C) residues are important for disulfide bond formation in human IL-15 (in human C^83^-C^133^ and C^90^-C^136^), and also conserved in fish IL-15 homologues. A phylogenetic tree was constructed using the known fish IL-15L protein sequences and the sequences of selected IL-2 family members from higher vertebrates (**Figure 3**). Phylogenetic tree shows that *Cc*IL-15L and IL-15L of other fish were clustered into a clade.

**Table 2:** Protein homology of IL-15L between common carp and other species.

Species	Molecules	Identity (%)	NCBI accession number
		*Cc*IL-15L	
*Cyprinus carpio*	IL-15L	100	OQ981449
*Pimephales promelas*	IL-15L	64.3	XP_039527011.1
*Puntigrus tetrazona*	IL-15L	60.1	XP_043114717.1
*Danio rerio*	IL-15L	53.2	XP_005157574.1
*Ictalurus punctatus*	IL-15L	33.5	XP_017345806.1
*Oncorhynchus mykiss*	IL-15L	26.8	CDM74103.1
*Oncorhynchus keta*	IL-15L	25.1	XP_035627921.1
*Salmo salar*	IL-15L	22.7	XP_045580359.1
*Oncorhynchus mykiss*	IL-15	14.6	XP 021430745.2
*Salmo salar*	IL-15	14.2	NP 001265994.1
*Ctenopharyngodon idella*	IL-15	13.9	QCE20959.1
*Homo sapiens*	IL-15	15.8	AAI00964.1
*Mus musculus*	IL-15	14.2	AAH23698.1

### 3.2 Analysis of *Cc*IL-15L expression in fish

**Figure 4:**
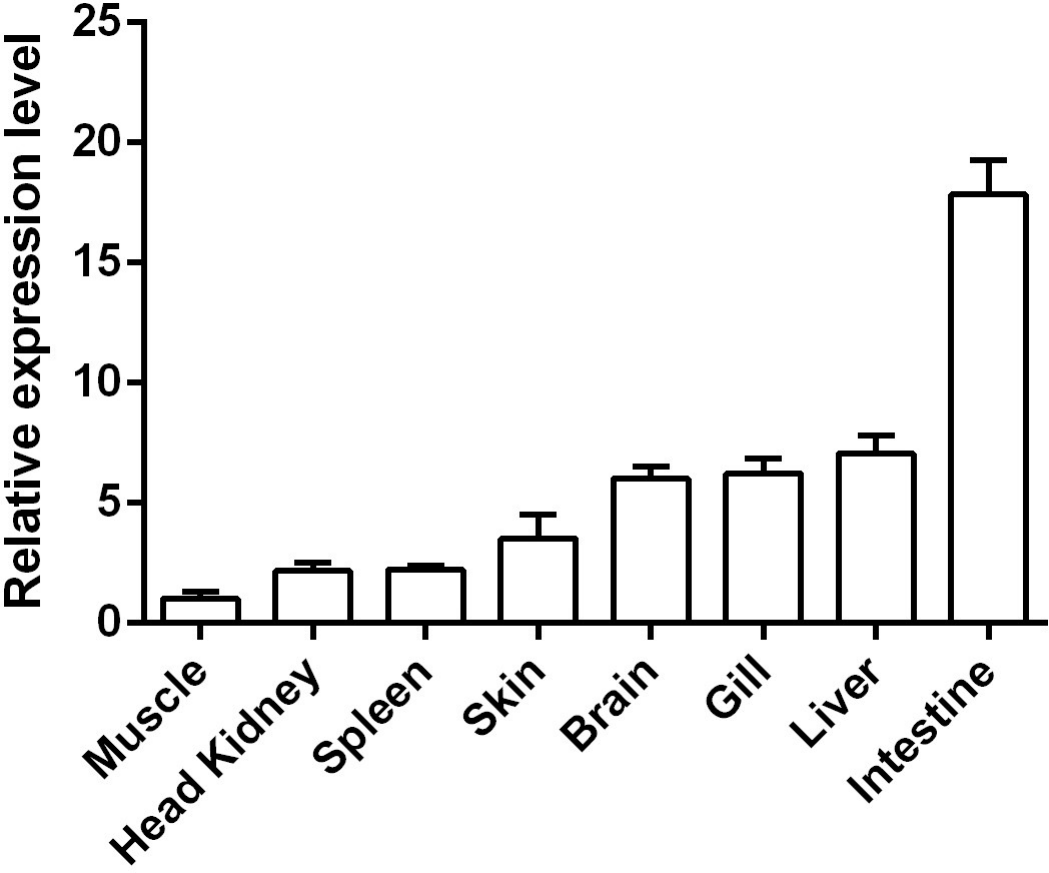
The expressions of *Cc*IL-15L in various tissues of healthy common carp. The mRNA expression levels of *Cc*IL-15L were normalized by *EF-1α* and expressed as the ratio of the expression levels in muscle. Data are shown as mean + SEM (n=5).

The relative expression levels of the *Cc*IL-15L gene in different tissues and organs were detected by RT-qPCR. As shown in **Figure 4**, *Cc*IL-15L is constitutively expressed at different levels in all tested tissues and organs, including the spleen, head kidney, skin, gills, brain, liver, intestine, and muscle. The intestine showed the highest level of expression, followed by the liver, gills, brain, and skin, with lower levels of expression in the spleen, head kidney, and muscle.

**Figure 5:**
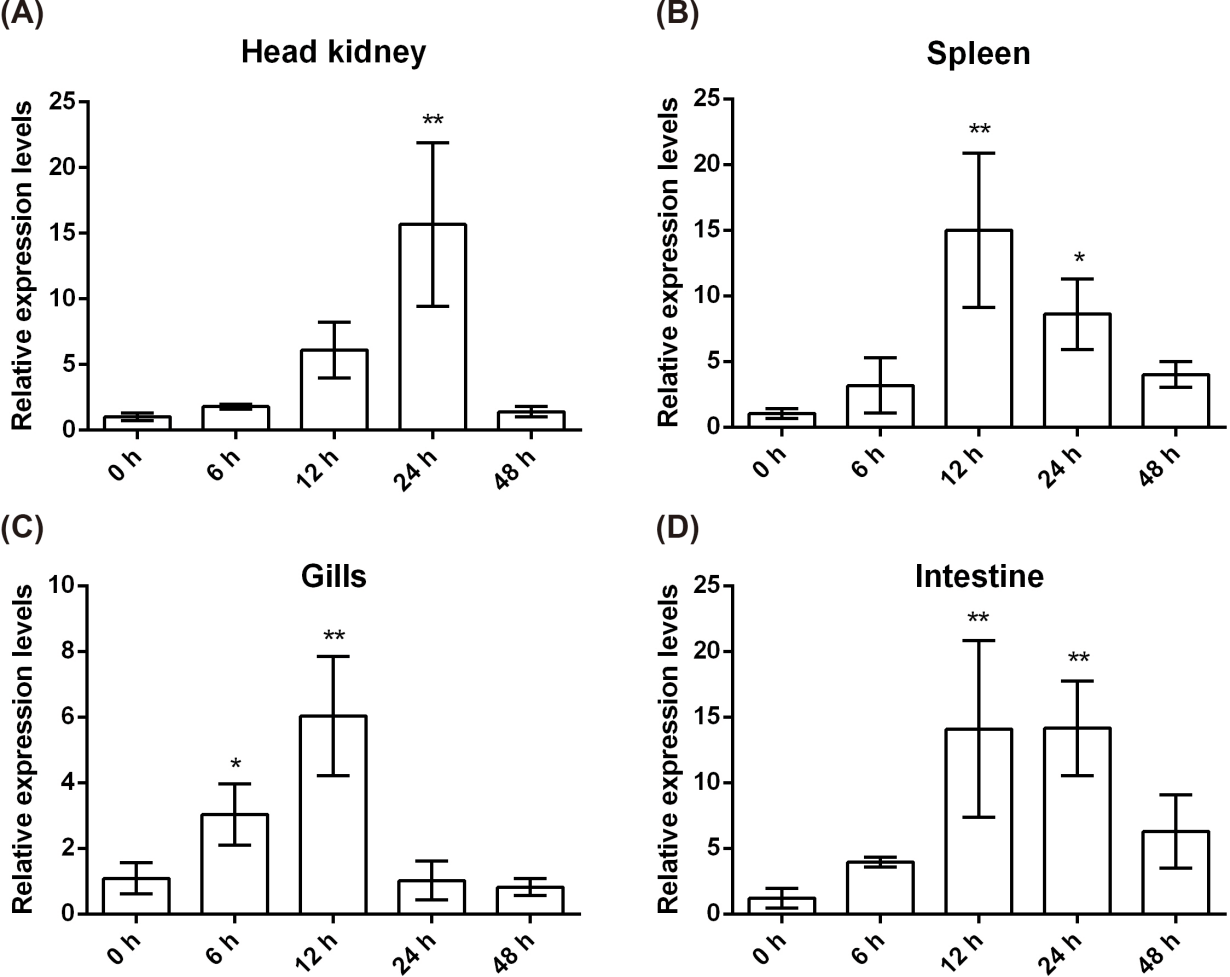
*Cc*IL-15L expression patterns at mRNA levels in tissues of common carp after infected with *A. hydrophila*. The different tissues include head kidney **(A)**, spleen **(B)**, gills **(C)**, and intestine **(D)**. Data are shown as mean + SEM (n=3). The *EF-1α* gene was used as an internal control. The significant difference was analyzed by comparing with the corresponding value at 0 h (“*” signs *p* < 0.05, and “**” signs *p* < 0.01).

To assess the *Cc*IL-15L response to bacterial infection, the fish were injected with *A. hydrophila*. Head kidney, spleen, gills, and intestine were sampled at 0, 6, 12, 24, and 48 h post injection (hpi) for examining gene expression. As shown in **Figure 5A**, in head kidney, the expression levels spiked at 24 h after injection but then returned to normal values by 48 h. In the gills and spleen, the expression levels were the highest at 12 hpi (**Figures 5B, C**). In the intestine, the expression level is also the highest at 12 hpi, and it is basically the same as the expression level at 24 hpi (**Figure 5D**).

### 3.4 Analysis of bioactivity of r*Cc*IL-15L protein

**Figure 6:**
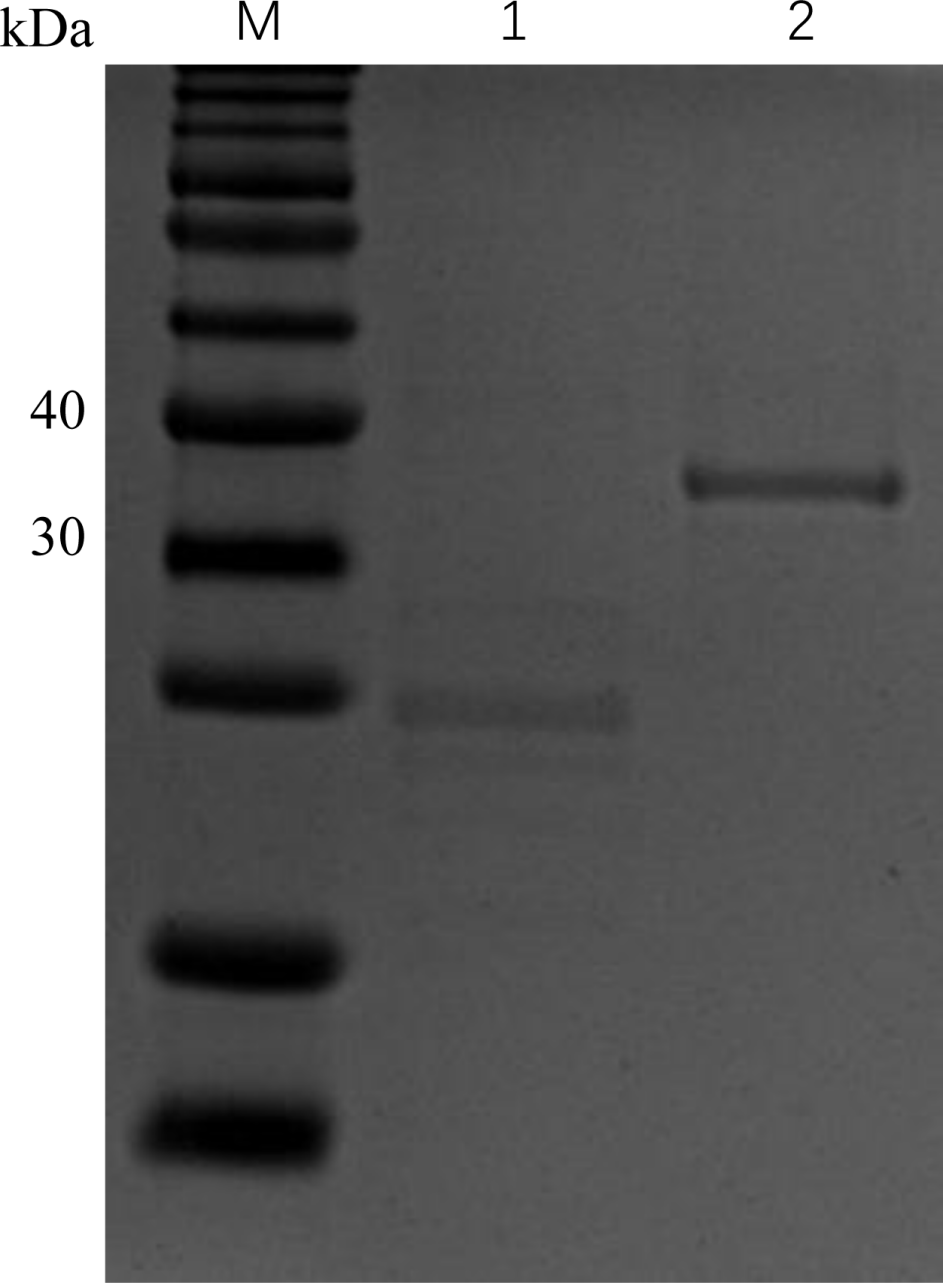
Expression of r*Cc*IL-15L and Trx proteins based on SDS-PAGE analysis. Lane M: protein marker; Lane 1: the Trx protein, Lane 2: the purified r*CcI*L-15L protein.

**Figure 7:**
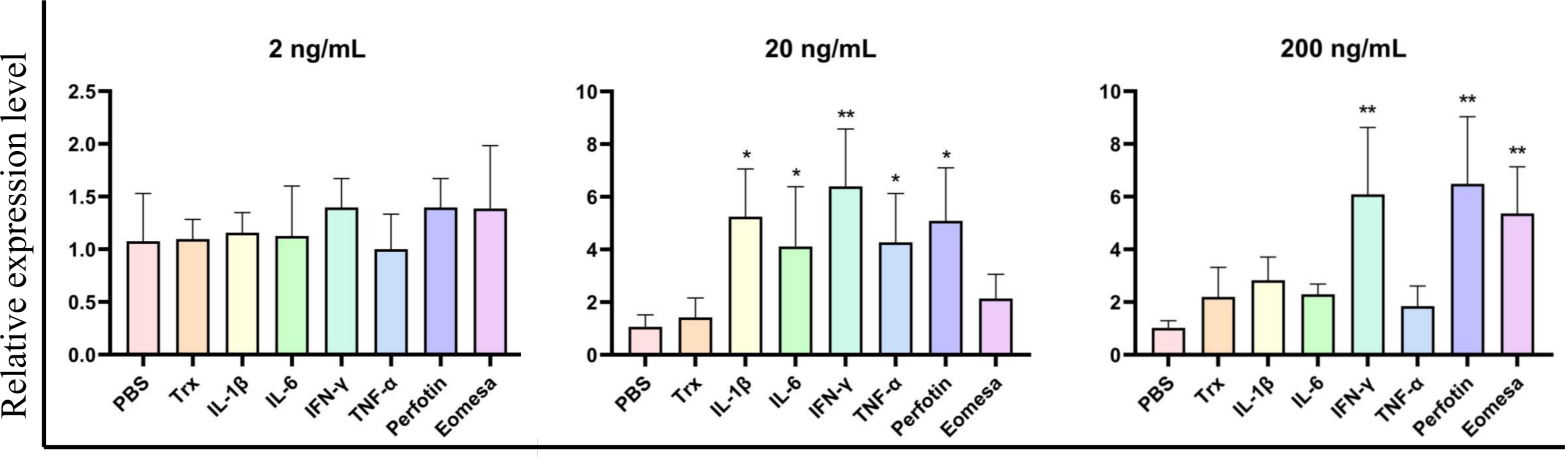
The effects of r*Cc*IL-15L on gene expression in the primary head kidney leukocytes. The primary head kidney leukocytes were stimulated with r*Cc*IL-15L for 12 h. The *EF-1α* gene was used as an internal control. Data are shown as mean ± SEM (n=3). The significant difference was analyzed by comparing with the corresponding value in the control (“*” signs *P* < 0.05, “**” signs *P* < 0.01).

We expressed and purified the r*Cc*IL-15L protein (35 kDa) in *E. coli* to determine its function (**Figure 6**). Then, we stimulated primary head kidney leukocytes with the r*Cc*IL-15L protein to investigate its effects on immune gene expression, including NK cell activation factors (*Perforin* and *Eomesa*) and pro-inflammatory cytokines (*IL-1β*, *IL-6*, *IFN-γ*, and *TNF-α*). As shown in **Figure 7**, when the leukocytes were stimulated with 2 ng/ml r*Cc*IL-15L protein, there was no significant increase at gene expression levels for six cytokines. However, when stimulated with 20 ng/ml protein, five cytokine expressions were significantly increased, and only gene *Eomesa* expression was not significantly upregulated. When stimulated with higher concentrations of r*Cc*IL-15L protein (200 ng/ml), NK cell activation genes (*Eomesa* and *perforin*) and *IFN-γ* expression were significantly up-regulated, while *IL-6*, *IL-1β*, and *TNF-α* gene expressions did not show significant up-regulation.

### 3.5 The effects of *Cc*IL-15L over-expression on immune-related genes and histopathological change *in vivo*

**Figure 8:**
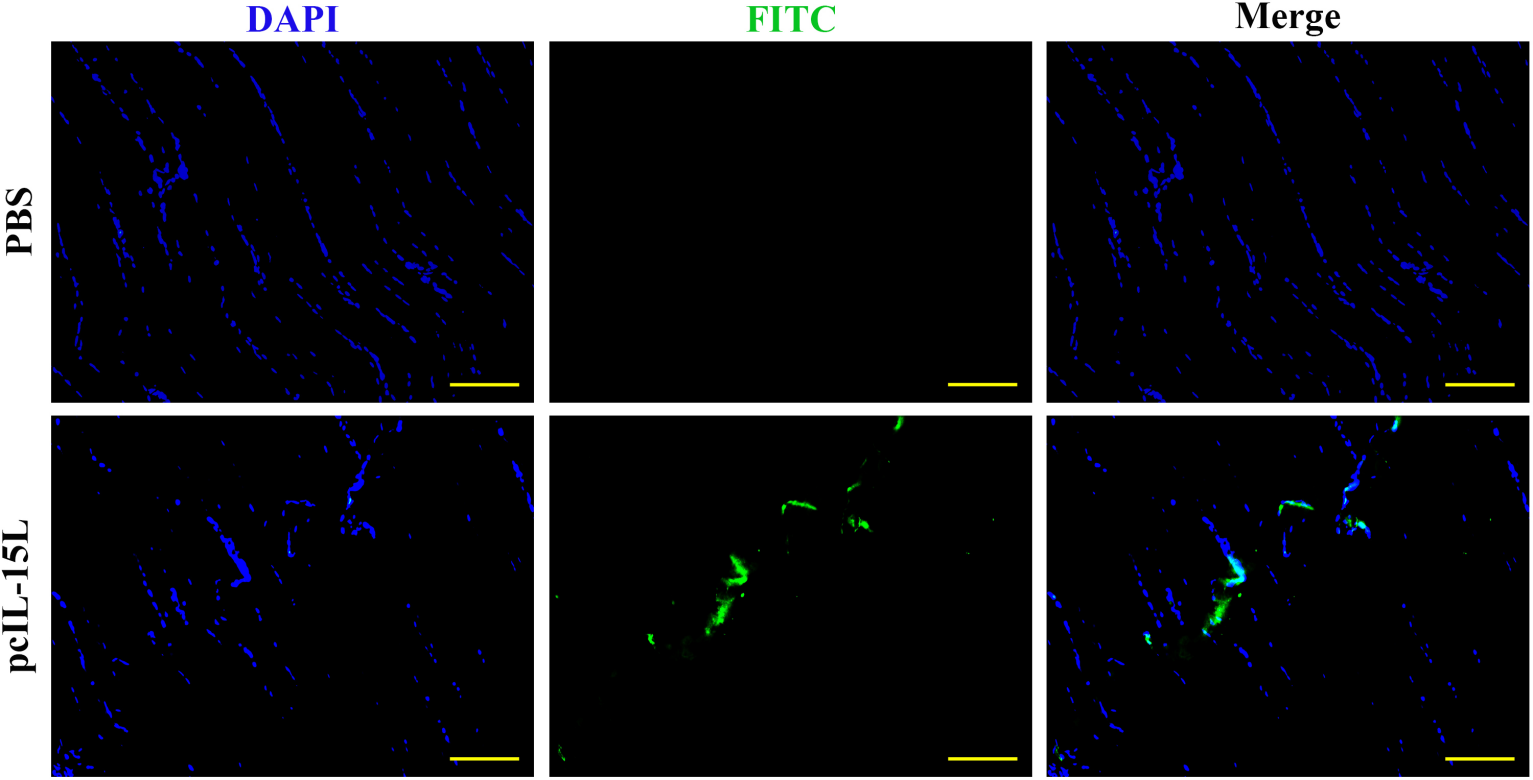
The expression of FLAG-tagged rIL-15L protein in the muscle tissues at 5 d after intramuscular injection was confirmed by indirect immunofluorescence. Indirect immunofluorescence was performed to detect the FLAG-tagged rIL-15L protein in muscle at 5 d after intramuscular injection, and the green part represents the region to express FLAG-tagged rIL-15L protein. Scale bars = 100 7μm.

**Figure 9:**
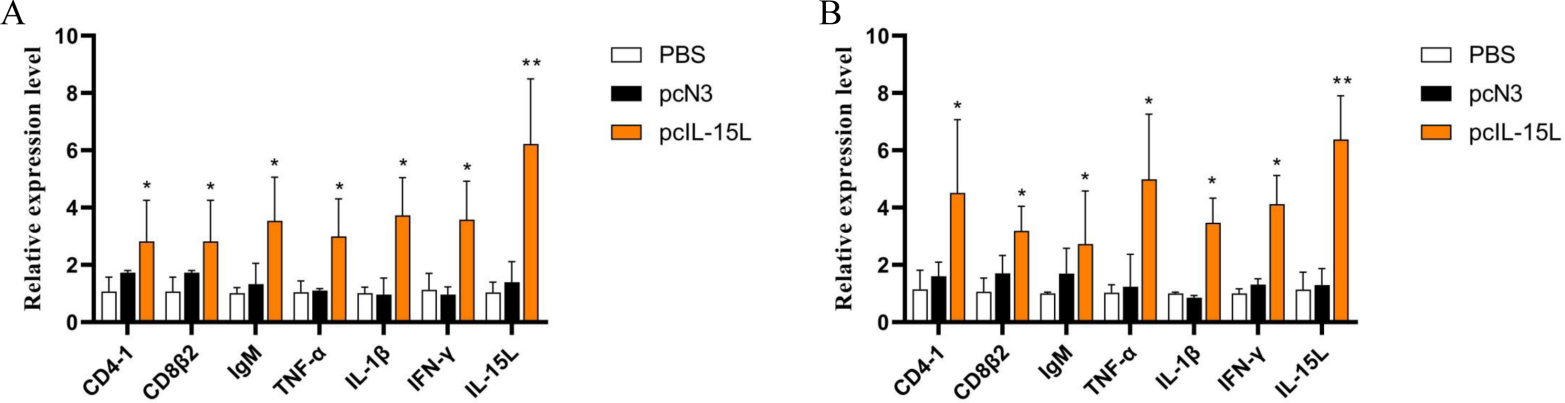
The effects of CcIL-15L overexpression on cytokine expression in common carp. The expressions of various cytokines in head kidney **(A)** and spleen **(B)** were detected by RT-qPCR at 5 d post plasmids injected. The *EF-1*α gene was used as an internal control. Data are shown as mean ± SEM (n=3). The significant difference was analyzed by comparing with the corresponding value in the control (“*” signs *P* < 0.05, “**” signs *P* < 0.01).

The results described above showed that the recombinant *Cc*IL-15L indicated regulatory activity *in vitro*. To further study the effect of *Cc*IL-15L *in vivo*, we constructed the pcIL-15L expressing vector, which could overexpress in common carp. As shown in **Figure 8**, based on IIFA methods, it was showed that the proteins of 3×FLAG-tagged pcIL-15L were detected in the muscle of common carp after injected with pcIL-15L plasmids for 5 d. By contrast, no FLAG-labeled signal was detected in the control. Meanwhile, RT-qPCR detection revealed that the expression of *Cc*IL-15L significantly increased in head kidney and spleen. Except for *Cc*IL-15L, immune-related genes including *IL-1β*, *CD4-1*, *CD8β2*, *IFN-γ*, and *TNF-α* were also significantly increased. In contrast, for these genes, there was no significant difference at the mRNA expression levels, in the PBS and pcN3 groups (**Figures 9A, B**).

**Figure 10:**
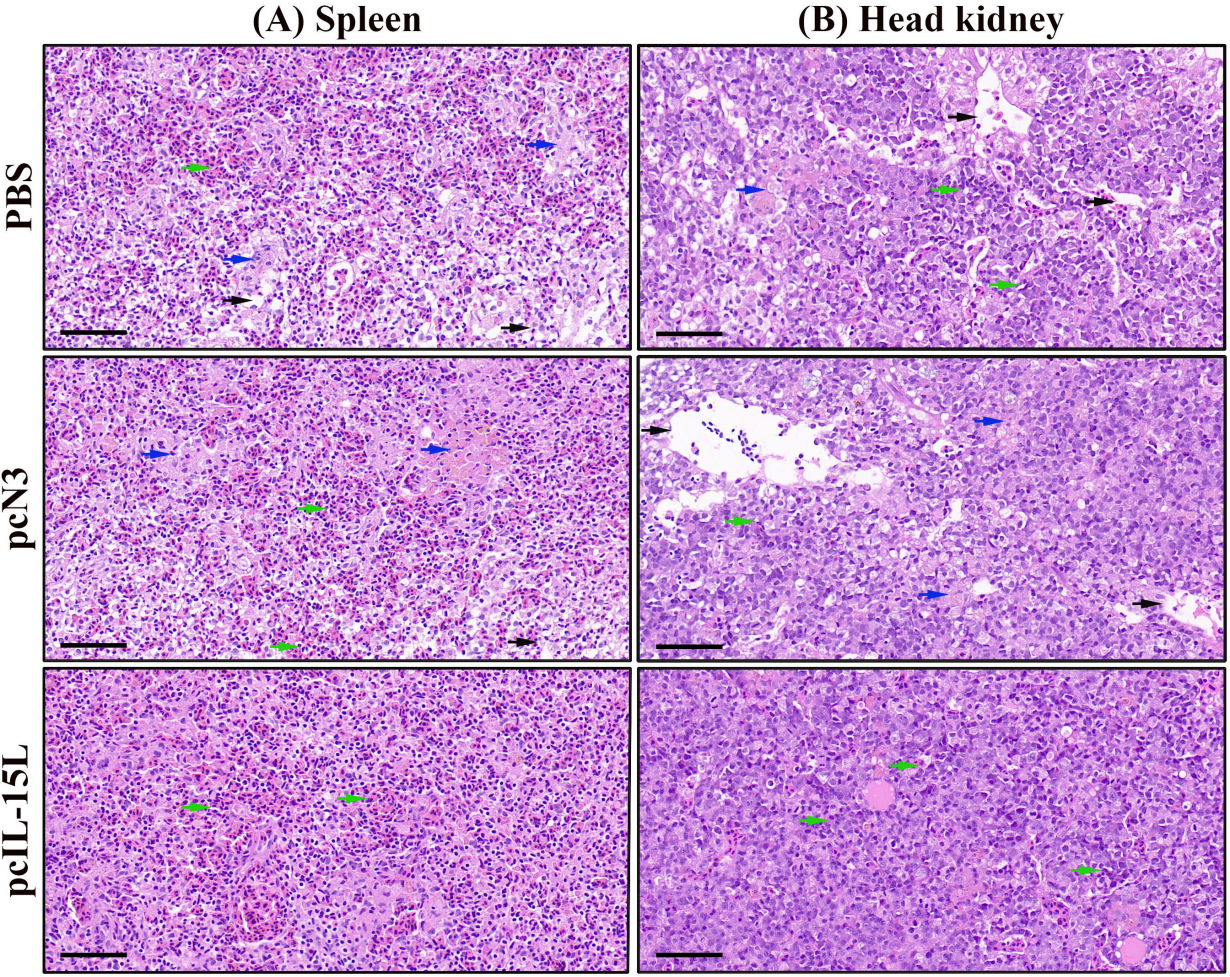
Histopathological changes in spleen **(A)** and head kidney **(B)** by HE staining. Histopathological lesions were shown with different color arrows such as tissue fibrosis (blue arrow), lymphocytic infiltration (green arrow), and tissue damage (black arrow). Scale bars = 50 μm.

The histopathological change was further evaluated in head kidney and spleen of common carp injected with pcIL-15L vectors after *A. hydrophila* challenge. As shown in **Figure 10**, the results showed that, compared with the group injected with pcIL-15L plasmid, histopathological lesions in head kidney and spleen showed to be more severe in the PBS and pcN3 group at 24 h, such as tissue fibrosis, lymphocyte infiltration, and tissue damage.

### 3.6 Effect of r*Cc*IL-15L overexpression on bacterial infection *in vivo*

**Figure 11:**
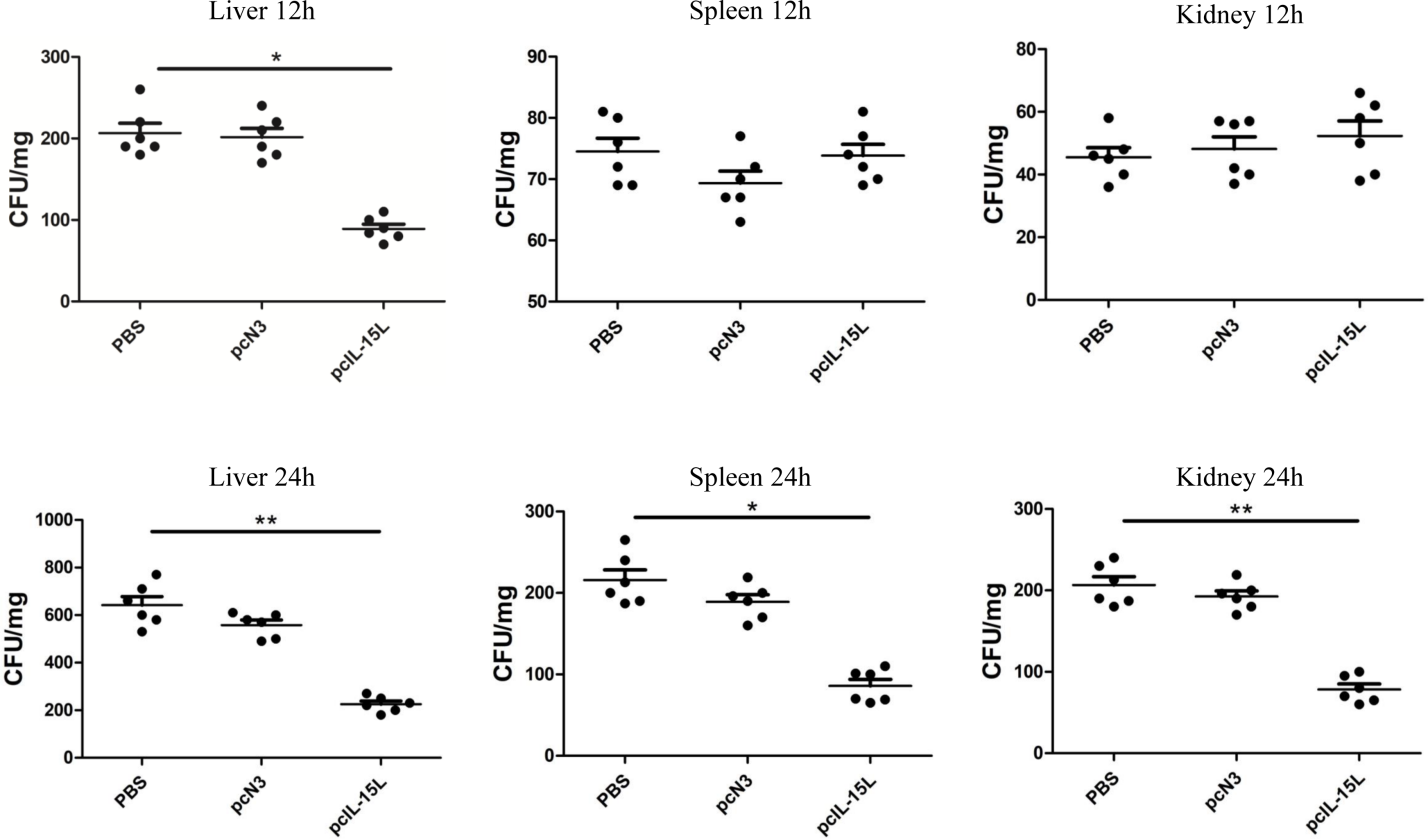
Bacterial loads in the liver, spleen and kidney of pcIL-15L over-expression fish were determined at 12 and 24 hpi. Data are shown as mean ± SEM (n = 6). “*” *P*< 0.05 or “**” *P*<0.01 are considered significant.

Five days after intramuscular injection of the recombinant plasmids, carp were challenged with *A. hydrophila* to evaluate bacterial infection by quantifying bacterial loads in the liver, spleen, and kidneys at 12 and 24 h post-infection. As shown in **Figure 11**, at 12 h after infection, bacterial colonization in the liver was significantly reduced in the pcIL-15L group compared to the control group. By 24 h post-challenge, bacterial loads in the liver, spleen, and kidney were markedly lower in the pcIL-15L group than in the controls.

### 3.7 The r*Cc*IL-15L promoted the phagocytosis and chemotactic abilities of HKLs

**Figure 12:**
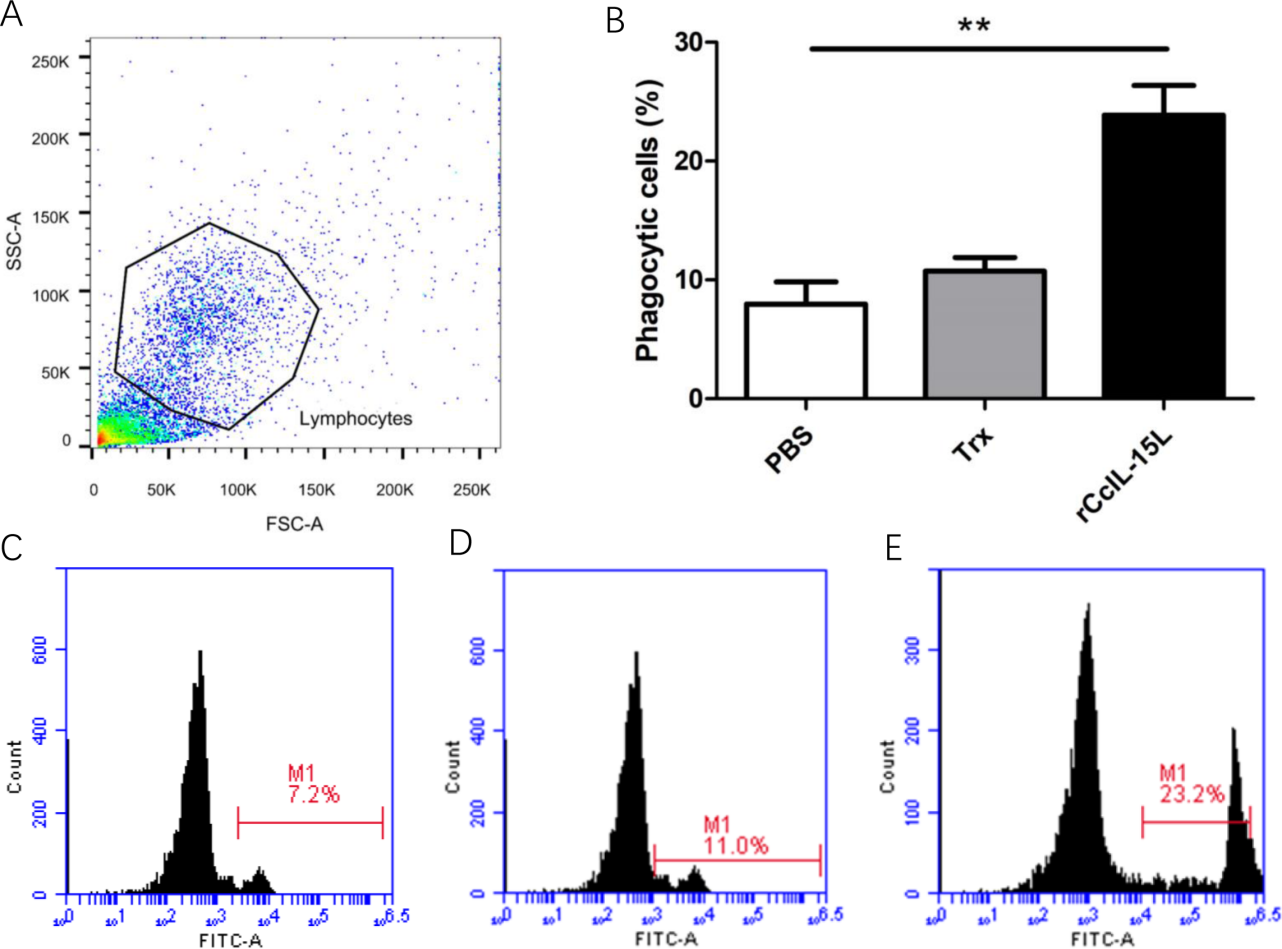
Recombinant *Cc*IL-15L enhanced the phagocytic activity of HKLs. **(A)** Flow cytometry was used for HKL cell typing. Common carp HKLs were treated with either r*Cc*IL-15L or PBS as a control. Following incubation with 1.0 μm fluorescent beads for 3 h, the phagocytic activity of HKLs was measured using flow cytometry. **(B)** Statistical evaluation of the leukocyte phagocytosis rate against fluorescent microspheres was conducted. **(C–E)** The fluorescence histogram illustrates the proportion of phagocytic leukocytes (M1 region) among the isolated leukocyte population. Representative data from a single fish are displayed.

After treatment of common carp head kidney leukocytes HKLs with r*Cc*IL-15L, phagocytic activity was assessed through flow cytometry (**Figure 12**). Lymphocyte populations were identified within the gated HKLs (**Figure 12A**). Stimulation with r*Cc*IL-15L significantly enhanced the phagocytic activity of HKLs, demonstrating its role in promoting immune function in these cells. (*P* < 0.01) (**Figures 12B–E**).

**Figure 13:**
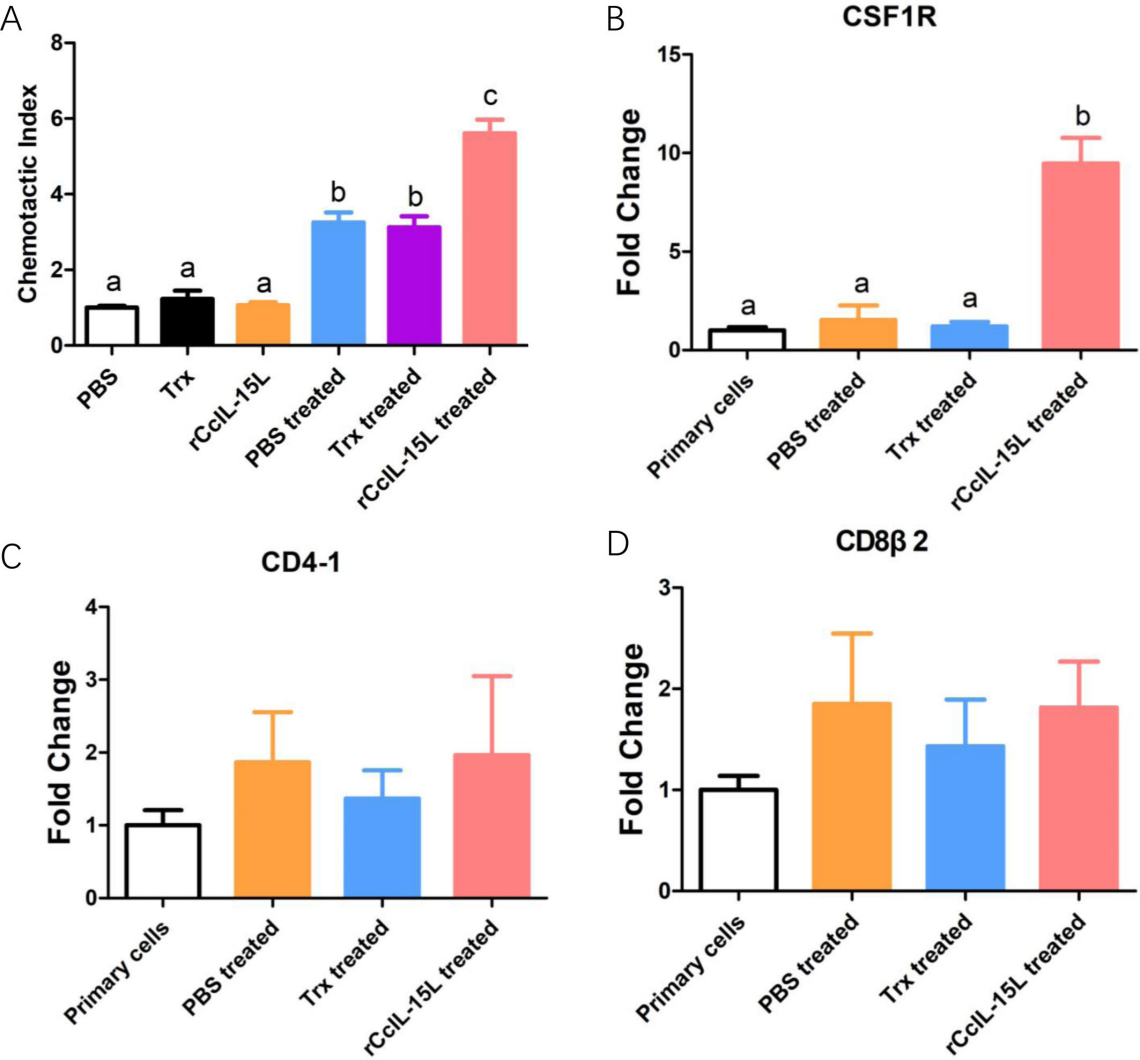
Recombinant *Cc*IL-15L elicited distinct chemotaxis responses of HKLs. **(A)** The migrated HKLs in response to PBS, Trx, r*Cc*IL-15L, Trx treated culture mediums or r*Cc*IL-15L treated culture mediums divided by the number of cells that migrated to the PBS. **(B–D)** The mRNA expression levels of markers for different cell types were detected by qPCR in migrated cells. Primary cells were used as a control. Different letters above the bar indicate significant differences.

To further investigate the chemotactic role of r*Cc*IL-15L, we assessed its capacity to attract common carp HKLs using a chemotaxis chamber. The findings revealed that while r*Cc*IL-15L alone did not enhance HKL migration, the culture supernatants from HKLs treated with either PBS, Trx or r*Cc*IL-15L significantly promoted the migration of HKLs (**Figure 13A**). We analyzed the expression levels of cell type marker genes using qPCR. The results indicated that the expression levels of macrophage marker CSF1R were significantly upregulated in the chemotaxed cells of r*Cc*IL-15L treated medium supernatants compared to both primary cells and the medium supernatants treated by PBS (**Figure 13B**). However, there were no significant differences in the expression of the lymphocyte markers CD4-1 and CD8β2 among the groups (**Figures 13C, D**).

## 4 Discussion

In this study, an IL-15 homologue in *C. carpio* (IL-15 like, termed as *Cc*IL-15L) was identified. The deduced IL-15L protein has a typical IL-15 domain (amino acid:49-157), including four cysteine residues to form two intrachain disulfides bonds, which play a key role in IL-15 binding to receptors (25). In addition, we found that IL-15L lacks a signal peptide, which was presumably secreted through the same non-classical pathway as IL-1, but not in the ER-Golgi pathway (26). *Cc*IL-15L indicated a high level of sequence identity with homologs from fathead minnow (64.3%) and tiger barb (60.1%), but a low level of sequence identity with other fish and mammals (10.6-33.5%). The phylogenetic tree showed that *Cc*IL-15L and other fish IL-15L were clustered into a major branch, indicating that *Cc*IL-15L is well conserved in teleost.

*Cc*IL-15L transcripts were constitutively expressed in all tested tissues. *Cc*IL-15L expression was the highest in the intestine, and it was speculated that *Cc*IL-15L was widely distributed in the intestinal tract and closely related to the occurrence and development of various intestinal diseases. Numerous studies in mammals have demonstrated that IL-15 plays a significant role in the intestine by encouraging intestinal epithelial cell proliferation and differentiation, preserving the integrity of the intestinal epithelial barrier, and defending the intestine from pathogenic microorganisms and harmful substances (27–30). IL-15L expression in zebrafish was found to be higher in the spleen, heart, gonads, skin, and gills, while weaker expression was observed in the thymus, head kidney, intestine, and liver. Notably, there was no expression detected in PBL and muscle (11). In trout, IL-15Lb expression was relatively high in the gills, and both IL-15La and IL-15Lb expression was relatively low in the head kidneys (20). The difference in the results might be due to difference in detection methods or species specificity.

Healthy common carp were artificially infected with *A. hydrophila* to investigate whether *Cc*IL-15L is involved in the immune response to bacterial infection. As shown in **Figure 5**, *Cc*IL-15L expression was significantly increased in the gills, intestine, head kidney, and spleen of bacterial-infected common carp. These findings are from previous studies conducted on other fish species, such as grass carp, rainbow trout, rock bream (*Oplegnathus fasciatus*), and dojo loach (*Misgurnus anguillicaudatus*). Furthermore, these studies also showed that pathogens significantly induced the expression levels of IL-15L both *in vivo* and *in vitro* (12, 14, 15, 31). PHA, PMA, and poly(I:C) could induce the expression levels of IL-15 in grass carp HKLs and splenocytes (15). In dojo loach, *Flavobacterium columnare* G4, *Ichthyophthirius multifil*e and *Saprolegnia parasitica* infections could significantly induced the expression level of IL-15. Therefore, these findings provide the supporting evidence that IL-15L is potentially involved in the antimicrobial immune response in fish.

In mammals, IL-15 regulates apoptosis and inflammatory responses and plays an important role in tumor immune surveillance (32, 33). In teleost, the studies on the biological activity of IL-15 homologue have been reported only in grass carp and rainbow trout (15, 20). In grass carp, the biological activity of r*Ci*IL-15 protein was evaluated in primary leukocytes, and r*Ci*IL-15 was found to induce type 1 immune responses (*IFN-γ* and *T-bet*) and signature genes for NK cell activation (*perforin* and *Eomesa*), while exhibiting inhibitory effects on the genes involved in type 2 immune responses (*IL-4/13*, *IL-10*, and *Gata3*) (15). Rainbow trout were used to test the biological activity of the rIL-15 protein in primary leukocytes, and it was found that rIL-15 significantly increased the levels of *CD4-1*, *CD8β2*, *IgM*, *IL-4/13*, *IFN-γ*, and *perforin* expression (20). In the current study, it was found that r*Cc*IL-15L could induce the expression levels of pro-inflammatory cytokines (*IL-1β*, T*NF-α*, *IFN-γ*, and *IL-6*) and activate NK cells (*perforin* and *Eomesa*). These findings suggest that *Cc*IL-15L could play a crucial role in promoting inflammatory responses and defending the host against pathogen infections.

In mammals, the functions and activities of IL-15 have been studied in depth. Among these immune functions, IL-15 can stimulate the proliferation/activation of CD4^+^ and CD8^+^ T cells and has preclinical antitumor activity (1, 34–37). Furthermore, IL-15 plays an important role in NK cell development, proliferation, and activation (38). IL-15 is also thought to critically regulate T helper cell differentiation and induce the expression of IgM and TNF-α (39, 40). In fish, the biological activity of rIL-15 still needs to be investigated to confirm its role in the immune response. In the present study, the constructed 3×FLAG eukaryotic expression plasmid encoding *Cc*IL-15L was successfully expressed in tissues of common carp, thereby inducing up-regulation of the expression levels of several immune-related genes. In this study, the expression levels of IgM significantly increased in spleens after pcIL-15L treatment, which indicated that IL-15L could defend bacterial pathogens through natural Abs. CD4-1 and CD8α are surface molecular markers of CD4^+^ and CD8^+^ T lymphocyte, respectively, which play an important role in antigen presentation and recognition, as well as immune signaling after pathogen invasion (41, 42). The up-regulation of CD4-1 and CD8α genes in the spleen suggests that pcIL-15L activates the function of CD4^+^ and CD8^+^ T lymphocytes. IL-15 signaling has been shown to activate the Th1 immune response by inducing the release of TNF-α (43, 44). In line with this finding, we observed that the expression levels of TNF-α obviously increased in spleen of common carp following pcIL-15L treatment, and it was confirmed that fish IL-15L could induced TNF-α and activate Th1 immune response. Additionally, we observed that common carp pre-treated with pcIL-15L exhibited higher immune protection levels against *A. hydrophila* infection, which was probably due to the strong immune response activated by pcIL-15L. This phenomenon may be related to the wide-ranging regulatory role and substantial effects of IL-15 on the cellular immune system. In mammals, IL-15 activates T cells, B cells, and NK cells and mediates the proliferation and survival of these cells, which has also been explored for therapeutic applications in different infections due to the important role of IL-15 in anti-tumor, pro-inflammatory, and anti-infection (40, 45).

In summary, an IL-15 homologue was identified in common carp. Expression of *Cc*IL-15L could be rapidly up-regulated in response to bacterial infection. The purified r*Cc*IL-15L protein exhibited a significant ability to induce the expression levels of inflammatory cytokines in HKLs. *In vivo* overexpression of *Cc*IL-15L was found to enhance the inflammatory response, indicating a substantial immune defense against bacterial infection. In this study, the role of *Cc*IL-15L was addressed in modulating immunity-related cytokines and in immune defense against pathogens.

## Data Availability

The datasets presented in this study can be found in online repositories. The names of the repository/repositories and accession number(s) can be found in the article/supplementary material.
